# Predicting clinically significant prostate cancer based on pre-operative patient profile and serum biomarkers

**DOI:** 10.18632/oncotarget.21297

**Published:** 2017-09-28

**Authors:** Izak Faiena, Sinae Kim, Nicholas Farber, Young Suk Kwon, Brian Shinder, Neal Patel, Amirali H. Salmasi, Thomas Jang, Eric A. Singer, Wun-Jae Kim, Isaac Y. Kim

**Affiliations:** ^1^ Section of Urologic Oncology, Rutgers Cancer Institute of New Jersey and Division of Urology, Rutgers Robert Wood Johnson Medical School, New Brunswick, NJ, USA; ^2^ Department of Biostatistics, Rutgers School of Public Health, Piscataway, NJ, USA; ^3^ Divison of Biometrics, Rutgers Cancer Institute of New Jersey, New Brunswick, NJ, USA; ^4^ Department of Urology, Chungbuk National University College of Medicine, Cheonju, Korea

**Keywords:** prostate cancer, biomarkers, prostate acid phosphatase

## Abstract

Previous studies have reported association of multiple preoperative factors predicting clinically significant prostate cancer with varying results. We assessed the predictive model using a combination of hormone profile, serum biomarkers, and patient characteristics in order to improve the accuracy of risk stratification of patients with prostate cancer. Data on 224 patients from our prostatectomy database were queried. Demographic characteristics, including age, body mass index (BMI), clinical stage, clinical Gleason score (GS) as well as serum biomarkers, such as prostate-specific antigen (PSA), parathyroid hormone (PTH), calcium (Ca), prostate acid phosphatase (PAP), testosterone, and chromogranin A (CgA), were used to build a predictive model of clinically significant prostate cancer using logistic regression methods. We assessed the utility and validity of prediction models using multiple 10-fold cross-validation. Bias-corrected area under the receiver operating characteristics (ROC) curve (bAUC) over 200 runs was reported as the predictive performance of the models. On univariate analyses, covariates most predictive of clinically significant prostate cancer were clinical GS (OR 5.8, 95% CI 3.1–10.8; *P* < 0.0001; bAUC = 0.635), total PSA (OR 1.1, 95% CI 1.06–1.2; *P =* 0.0003; bAUC = 0.656), PAP (OR 1.5, 95% CI 1.1–2.1; *P =* 0.016; bAUC = 0.583), and BMI (OR 1.064, 95% C.I. 0.998, 1.134; *P* < 0.056; bAUC = 0.575). On multivariate analyses, the most predictive model included the combination of preoperative PSA, prostate weight, clinical GS, BMI and PAP with bAUC 0.771 ([2.5, 97.5] percentiles = [0.76, 0.78]). Our model using preoperative PSA, clinical GS, BMI, PAP, and prostate weight may be a tool to identify individuals with adverse oncologic characteristics and classify patients according to their risk profiles.

## INTRODUCTION

As the debate regarding the management of early prostate cancer (PCa) continues, PCa remains a common cause of death in many men. In 2015, there was an estimated 220,800 new cases with an estimated 27,540 deaths [1]. Despite some positive results from screening trials [2], there remains a concern for overdiagnosis and overtreatment of indolent tumors with its associated morbidity [3]. Active surveillance (AS), therefore, has emerged as a solution to overtreatment of PCa [3, 4]. However, the success of AS depends on our ability to identify patients who will not benefit from treatment, or to be able to identify important triggers for treatment without compromising oncologic outcomes [5, 6]. Various protocols are used at major institutions as eligibility criteria for entry into AS. These protocols are mostly centered on a combination of clinical stage, Gleason score, prostate-specific antigen (PSA), PSA density, number of positive prostate biopsies, and/or percent of malignant tissue present per core [7–11]. However, there is still a measurable risk of upstaging and upgrading of PCa, thus possibly missing the window of curative intervention [12, 13]. Numerous adjunct tests currently exist to help more accurately stratify patients preoperatively, though their true clinical utility in this setting is still unknown [14]. We therefore examined whether common preoperative variables in men with PCa who were otherwise candidates for AS, but underwent radical prostatectomy, were predictive of clinically significant PCa. This can potentially increase the accuracy of stratifying patients prior to recommending AS.

## MATERIALS AND METHODS

### Study cohort

After obtaining the approval from the Institutional Review Board (IRB), we conducted a retrospective analysis of a prospectively maintained database of men who underwent robotic assisted radical prostatectomy (RARP) at our comprehensive cancer center between January 2012 and May 2014. All patients were diagnosed with localized PCa via transrectal ultrasound–guided prostate biopsy with a minimum of 12 cores per biopsy. The attending urologist then assigned clinical stage according to the TNM system. All biopsy and RARP specimens were reviewed by a genitourinary pathologist. Patients with incomplete data were excluded from this study.

### Outcome variables

Patients were identified for analysis if they had complete data available on multiple variables and those include, patient characteristics (age, race, BMI), tumor characteristics (clinical stage, pre-operative GS) as well as preoperative serum biomarkers, which have been shown in previous studies to be predictive of advanced prostate cancer. Markers such as free PSA, total PSA, prostate weight as a surrogate of volume [15], PTH and calcium [16], acid phosphatase [PAP] [17], testosterone [18], and chromogranin A [CgA]) [19] were used. Patients whose post-op Gleason score was 4 + 3 or higher or post-op pathologic stage was non-organ confined were classified in high-risk, and otherwise low-risk. Patients with GS upgrading (i.e. from 3 + 3 or 3 + 4 to 4 + 3 or higher) or pathologic upstaging (i.e. from non-organ confined to organ confined) were considered to have clinically significant prostate cancer.

### Statistical analysis

**Figure 1:**
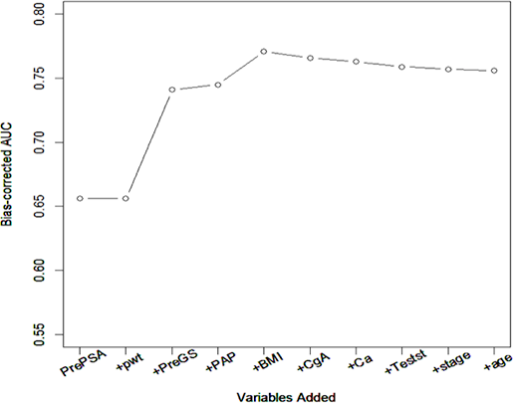
Sequential addition of predictive variables

Patients with unavailable or incomplete data were excluded from the analysis. We described baseline characteristics of patients who underwent RARP between 2012 and 2014. Twelve clinical factors were considered to build a predictive model: age (years), BMI, pre-operative clinical stage (T2–T3 vs. T1), pre-op Gleason score (low vs. high risk), pre-op PSA (mg/nl), prostate weight (grams), PTH pg/mL and Ca, PAP ng/mL, testosterone ng/dL, and CgA ng/mL. Univariate and multivariate logistic regression methods were employed to build a predictive model for high vs. low risk confirmed post-operatively. Area Under the ROC curve (AUC) was calculated for each of the models as a measurement of predictive performance. Model building strategy we adopted was as follows: (i) for each of predictors, a univariate logistic regression model was employed to calculate its AUC; (ii) Predictors were ranked based on their AUCs; (iii) Predictors were added to the model one at a time based on their AUC ranking Figure 1; and (iv) Two hundred runs of 10 fold-cross validation were implemented to calculate bias-corrected AUC [bAUC] (i.e. averaged AUC over 200 runs) for each of the models in (iii) as well as [2.5, 97.5] percentiles. A model with the best bAUC was determined as the final predictive model. All analyses were conducted using R 3.1.0.

## RESULTS

### Study cohort

There were 224 patients with complete data that were available for inclusion in this analysis. A total of 23 patients were excluded from the study due to incomplete data (15), neoadjuvant hormone therapy (1), metastatic PCa (1), clinical T3 disease (6). The majority of the study subjects were white men (82%), and mean age and BMI were 60.9 years, and 28.2, respectively. With regards to tumor characteristics, 68.8% of patients had low-grade disease (3 + 3 or 3 + 4), with the majority being low-clinical stage (non-palpable) 89.7% (Table 1). The rate of clinically significant prostate cancer post-operatively was 35%.

**Table 1:** Baseline study cohort characteristics

Variable		Total ( *n* = 224)
Age, Mean (SD) (years)		60.93 (6.86)
BMI, Mean (SD) (kg/m^2^)		28.23 (4.28)
Pre-op PSA, Mean (SD) (ng/ml)		6.98 (5.73)
Pre-op GS, N (%)	High (4 + 3/Higher)	70 (31.2)
	Low (3 + 3/3 + 4)	154 (68.8)
Clinical stage, N (%)	cT2	23 (10.3)
	cT1	201 (89.7)
Race, N (%)	Caucasian	184 (82.1)
	African-American	31 (13.8)
	Others	9 (4.1)
Prostate weight, Mean (SD) (gram)		50.39 (19.52)
PAP, Mean (SD) (ng/mL)		1.73 (1.03)
Testosterone, Mean (SD) (ng/dL)		417.13 (161.59)
CgA, Mean (SD) (ng/mL)		156.69 (239.19)
Calcium, Mean (SD) (ng/mL)		9.58 (0.41)
PTH, Mean (SD) (pg/mL)		46.78 (52.34)

### Outcome variables

Median values for the following pre-operative markers were PSA 5.2 (range 0.8–53.9), prostate weight 45.5 (20–151), PAP 1.5 (0.5–11), testosterone 399 (42–954), CgA 89 (21–1947), calcium 9.5 (8.5–11.2), and PTH 40 (15–780). Twelve predictors of interest were evaluated for predictive performance in univariate logistic regression approach, and results were listed in Table 2. Pre-op PSA showed the best performance with bAUC 0.656 with (2.5, 97.5) percentiles [0.646, 0.662]. High grade biopsy GS ( bAUC = 0.635 [0.609, 0.653]), PAP (bAUC = 0.583 [0.572, 0.591]), BMI (bAUC = 0.575 [0.562, 0.585], CgA (bAUC = 0.553 [0.538m 0.566] and Ca (bAUC = 0.537 [0.512, 0.553]) were ranked in order. Prostate weight (bAUC = 0.509 [0.481, 0.55]) was not predictive of post-op cancer status in univariate setting. Predictors were added one at a time to build a multivariate model. The order of addition was determined by their predictive performance in univariate setting as well as their clinical relevance. Even the univariate analysis was negative, BMI and prostate volume were included in the final multivariate analysis because these factors (prostate volume in the context of PSA density) have been reported to be associated with prostate cancer prognosis. Orders were as follows: pre-op PSA, prostate weight, pre-op GS, PAP, BMI, CgA, Ca, testosterone, pre-op clinical stage, and age. Plot of bAUC and each model is displayed in Figure 1. As seen in Figure 1, bAUCs have plateaued out when BMI was added to the model with pre-op PSA, prostate weight, pre-op GS status, and PAP, and reached to its maximum value at 0.771 [0.76, 0.78]. The addition of the remaining variables to the model did not increase predictive performance further (Table 3). The estimates of the final model are summarized in Table 4.

**Table 2:** Univariate logistic regression measuring predictive performance of variables

Variable	OR (95% C.I.)	*p* -value	AUC (original data)	Bias-corrected AUC & [2.5, 97.5] percentiles
Age (years)	1.021	0.315	0.54	0.513
	[0.981, 1.063]			[0.487, 0.532]
Height (inch)	1.056	0.242	0.553	0.537
	[0.964, 1.158]			[0.522, 0.551]
Weight (lb)	1.008 [1.001, 1.017]	0.038	0.605	0.595
				[0.584, 0.602]
BMI (kg/m2)	1.064	0.056	0.589	0.575
	[0.998, 1.134]			[0.562, 0.585]
Clinical stage	3.254	0.009	0.558	0.517
(cT1 vs. cT2)	[1.357, 8.186]			[0.5, 0.533]
Pre-op GS	5.754	< 0.0001	0.689	0.635
(3 + 3/3 + 4 vs. 4 + 3/higher)	[3.144, 10.768]			[0.609, 0.653]
Pre-PSA (ng/ml)	1.132	0.0003	0.664	0.656
	[1.063, 1.216]			[0.646, 0.662]
Complex PSA (ng/ml)	1.142	0.0003	0.668	0.661
	[1.068, 1.235]			[0.653, 0.666]
Prostate weight (gram)	0.995	0.511	0.523	0.509
	[0.98, 1.009]			[0.481, 0.55]
PTH (pg/mL)	0.999	0.671	0.519	0.513
	[0.988, 1.004]			[0.465, 0.56]
PAP (ng/mL)	1.497	0.016	0.592	0.583
	[1.108, 2.131]			[0.572, 0.591]
Testosterone (ng/dL)	0.999	0.316	0.544	0.523
	[0.997, 1.001]			[0.509, 0.424]
CgA (ng/mL)	0.999	0.132	0.567	0.553
	[0.997, 1.0001]			[0.538, 0.566]
Calcium (ng/mL)	1.431	0.291	0.554	0.537
	[0.735, 2.808]			[0.512, 0.553]

**Table 3:** Bias-corrected area under a receiver operation characteristic curve in predictive model building

Order	Predictors	Bias-corrected AUC [2.5, 97.5] percentile
1	PrePSA	0.656 [0.646, 0.662]
	PrePSA + Prostate weight	0.656 [0.641, 0.665]
2	PrePSA + Prostate weight + PreGS	0.741 [0.729, 0.75]
3	PrePSA + Prostate weight + PreGS + PAP	0.745 [0.735, 0.754]
4	PrePSA + Prostate weight + PreGS + PAP + BMI	0.771 [0.76, 0.78]
5	PrePSA + Prostate weight + PreGS + PAP + BMI + CgA	0.766 [0.755, 0.774]
6	PrePSA + Prostate weight + PreGS + PAP + BMI + CgA + Ca	0.763 [0.748, 0.775]
7	PrePSA + Prostateweight + PreGS + PAP + BMI + CgA + Ca + Testosterone	0.759 [0.745, 0.772]
8	PrePSA + Prostate weight + PreGS + PAP + BMI + CgA + Ca + Testosterone + Clinical stage	0.757 [0.746, 0.769]
9	PrePSA + Prostate weight + PreGS + PAP+BMI + CgA + Ca + Testosterone + Clinical stage + Age	0.756 [0.739, 0.768]

Abbreviations: PrePSA: Preoperative PSA; PreGS: Preoperative Gleason score; PAP: Prostatic Acid phosphatase; BMI: Body mass index; CgA: Chromagranin A; Ca: Calcium.

**Table 4:** Multivariate logistic regression model predictive of clinically significant prostate cancer

	Estimate (SE)	OR (95% C.I.)	*P*-value
PrePSA	0.11 (0.04)	1.117 [1.033, 1.207]	0.005
Prostate weight	−0.021 (0.01)	0.979 [0.96, 0.999]	0.039
PreGS	1.609 (0.34)	4.997 [2.592, 9.635]	< 0.0001
PAP	0.429 (0.2)	1.536 [1.033, 2.283]	0.034
BMI	0.101 (0.04)	1.106 [1.027, 1.19]	0.008

Abbreviations: SE: Standard error; PrePSA: Preoperative PSA; PreGS: Preoperative Gleason score; P^1, 2^AP: Prostatic acid phosphatase; BMI: Body mass index.

## DISCUSSION

There are multiple factors that contribute to the selection of men for AS in very-low risk prostate cancer (VLPCa). These factors relate to patient characteristics, biopsy features, serum markers and hormones, and genetic profile. Various combinations of these factors are currently used in risk stratifying patients prior to recommending AS with variable success. Patient characteristics such as race, age, family history and even BMI have been studied as predictors of patients who are at high risk of harboring or developing advanced PCa. For example, Sundi et al. and Ha et al. found that African-American (AA) men with a VLPCa were at significantly higher risk of upgrading and upstaging [20], whereas Jalloh et al. did not find a significant difference among racial groups despite AA men having higher rate of positive surgical margins in their cohort [21]. Age on the other hand has been found to be an important factor in predicting high risk PCa whereas BMI and family history have had conflicting results [14]. Biopsy features have been studied extensively, and found that Gleason Score (GS), number of positive cores and volume of disease were important predictors for disease progression in men with VLPCa [14]. In addition, the incorporation of PSA and PSA derivatives in the decision making process has become indispensable, though the data on its accuracy is mixed. Recently, genomic discoveries have led to the development of a plethora of genomic markers which aid in risk stratifying PCa patients such as prostate cancer antigen 3 (PCA3) and transmembrane protease, serine avian erythroblastosis virus E26 oncogene homolog gene fusions (TMPRSS2:ERG) [22]. However, results are also variable and the usefulness of these markers remains to be seen. Another useful metric in distinguishing pathologically indolent versus aggressive disease is the 4K test, a panel of four kallikrein markers: total prostate-specific antigen (PSA), free PSA, intact PSA, and kallikrein-related peptidase 2. This test has great predictive potential based on statistical modeling but as of yet its clinical utility remains unproven [23].

Characterization of a patient’s hormonal milieu may also play an important role in identifying high-risk PCa. There is increasing evidence that low preoperative testosterone levels are an independent predictor of various adverse PCa outcomes, including high-grade PCa, tumor bilaterality, risk of progression and biochemical recurrence, and disease upstaging leading to treatment initiation during active surveillance [24, 25]. Conversely, there is evidence showing a link between high testosterone levels and poor PCa outcomes, and so definitive conclusions cannot be drawn at this time [26]. Prostatic acid phosphatase (PAP) was the seminal biomarker used in the diagnosis of prostate cancer several decades ago, but was eventually supplanted by PSA due to its poor sensitivity in PCa screening and inferiority as an indicator of PCa recurrence. Recently, however, PAP has re-emerged as an important variable in identifying high-risk patients, as multiple studies have demonstrated that elevated pretreatment PAP predicts biochemical failure, clinical recurrence, metastatic disease, and overall survival [17]. Limited data suggesting a relationship between parathyroid hormone (PTH) and PSA exists, though the link is merely correlational and not established with any oncologic outcomes [16]. Chromogranin-A (CgA), commonly expressed in neuroendocrine tumors, may be associated with poorly differentiated PCa [27]. However, a large prospective analysis of prostate biopsy specimens showed no link between serum CgA and high-grade PCa, indicating that the predictive role CgA is currently indeterminate [19].

Knowing the potential preoperative factors that portend poor outcomes in PCa, we sought to create a multi-variable model to predict clinically significant PCa. Our univariate logistic analysis identified the variables most highly associated with post-prostatectomy Gleason score and non-organ confined disease. A multivariable logistic regression model was then constructed using the most predictive and clinically relevant preoperative factors, including: preoperative Gleason score, preoperative PSA, preoperative prostate weight, PAP, BMI, CgA, calcium, testosterone, clinical stage, and age. The final multivariate model with the best bias-corrected AUC (0.771) included only five preoperative variables: preoperative PSA, preoperative prostate weight, preoperative Gleason score, PAP, and BMI.

Our results indicate that a combination of common and easily accessible preoperative variables may prove useful in identifying those patients eligible for AS who are at a high risk of upgrading and/or upstaging. When determining if a patient is an appropriate candidate for AS, our model may serve as an additional piece of predictive information and aid in stratification into high versus low risk groups.

An interesting aspect of our study is that of the ten variables in the univariate analysis, PAP had the fourth best AUC, while in the multiple logistic regression model PAP was significantly associated with higher risk prostate cancer (*p* = 0.034). This suggests that PAP may be an underutilized serum marker and could play an important role in identifying patients harboring high risk PCa. This finding is in-line with previous data showing that PAP fares well with predicting systemic disease and overall disease recurrence. The proposed mechanism is that PAP predicts micro-metastatic spread before treatment initiation and thus is a prognostic marker for disease failure via distant tumor spread. [17]

There are several limitations to our study. First, we used post-prostatectomy prostate weight rather than preoperative prostate volume in our predictive model. It should be noted, however, that prostate weight is well established as a surrogate for prostate volume, as several papers have shown an excellent concordance between the two metrics [15, 28]. Another limitation is that our model is not externally validated. We did employ an internal validation method with multiple runs of 10-fold cross validation in order to avoid sampling bias. Further, we used pathologic outcome to define clinically significant cancer. In the future, we plan to carry out a validation study using the clinical course as the final outcome variable. Finally, our model and results are hypothesis generating and lay the foundation for other investigators to externally validate our model on outside populations.

## CONCLUSIONS

Our model using pre-operative PSA, clinical GS, BMI, PAP, and prostate weight was predictive of clinically significant prostate cancer and may be a tool to stratify patients with a diagnosis of prostate cancer with consideration being given to place the patient on active surveillance versus surgical treatment. Further studies with a larger cohort are needed to externally validate this model.

### Clincal practice point

Multiple preoperative risk assessment tools have been described to stratify patients with localized prostate cancer into different risk groups. This has become more important in the setting of active surveillance, a widely accepted management for patients with relatively lower risk profiles. In our study, we have incorporated both demographic and serum biomarkers as potential variables that might be associated with upgrading and upstaging. Our final multivariate model with the best bias-corrected AUC (0.771) included only five preoperative variables: preoperative PSA, preoperative prostate weight, preoperative Gleason score, PAP, and BMI. While preoperative PSA, prostate weight, Gleason score, and BMI have previously investigated parameters with regards to adverse pathologic characteristics, PAP has not been extensively reviewed. Our study findings suggest that PAP could play an important role in identifying patients harboring high risk PCa.

## Abbreviations

PCa: (prostate cancer)
RARP: (robot assisted radical prostatectomy)
PAP: (prostate acid phosphatase)
CgA: (chromogranin A)
BMI: (body mass index)
AUC: (area under curve)
PSA: (prostate specific antigen)
PCA3: (prostate cancer antigen 3)
